# Hyaluronic Acid Hydrogel Combined With Ocimum sanctum and Tendon-Derived Extracellular Matrix Enhances Tenogenesis in Periodontal Ligament Stem Cells

**DOI:** 10.7759/cureus.54060

**Published:** 2024-02-12

**Authors:** Aardra BS, Balaji Ganesh S, Rajalakshmanan Eswaramoorthy, Kaarthikeyan G

**Affiliations:** 1 Department of Periodontics, Saveetha Dental College and Hospitals, Saveetha Institute of Medical and Technical Sciences, Saveetha University, Chennai, IND; 2 Department of Biochemistry, Saveetha Dental College and Hospitals, Saveetha Institute of Medical and Technical Sciences, Saveetha University, Chennai, IND

**Keywords:** extracellular matrix, hyaluronic acid, ovine tendon, ocimum sanctum, tulsi leaf extract, periodontal regeneration, periodontal ligament stem cells

## Abstract

Background

*Ocimum sanctum* (OS) is a medicinal plant with antioxidant, anti-cancer, anti-diabetic, antimicrobial, and anti-inflammatory activities. Extracellular matrix (ECM) maintains the structural stability of tissues. Hyaluronic acid (HA), which is used in hydrogel fabrication and osteochondral regeneration, increases cell viability and the expression of marker genes. Periodontal ligament stem cells (PDLSC), which are a type of mesenchymal stem cells (MSC), have self-renewing capacity and they prevent teratoma formation and promote tendon (TEN) regeneration. The aim of this study is to incorporate the phytochemical effects of *Ocimum sanctum* into hyaluronic acid hydrogel scaffolds made with the MSCs in tendon ECM for increased tissue regeneration.

Materials & methods

*Ocimum sanctum* extract and methacrylated hyaluronic acid (HA-MA) were prepared. An ovine tendon sample was decellularised to obtain the ECM. The study groups of HA, TEN, HA_OS, HA_TEN, and HA_OS_TEN were prepared. The presence of tendon cells was confirmed by picrosirius red staining and the hydrogel scaffolds were analysed using scanning electron microscopy (SEM), swelling, differentiation, compression, and 3-(4,5-dimethylthiazol-2-yl)-2,5-diphenyl-2H-tetrazolium bromide (MTT) compatibility analyses.

Results

The morphology of the samples was analysed by SEM analysis. The HA_OS_TEN sample showed the highest rate of tenogenesis, lowest swelling, high cell viability and differentiation, and optimal compression rates.

Conclusion

This study showed that hyaluronic acid combined with *Ocimum sanctum* and tendon ECM is a very good conjugation for the preparation of hydrogel scaffolds for tendon tissue regeneration using MSCs.

## Introduction

Extracellular matrix (ECM) is the substance in a tissue in which the cellular components of the tissue are suspended. The important constituents of the ECM are endothelial cells, mast cells, and fibroblasts, which form the fibrous content of the tissue and release growth factors [1]. The role of the ECM is to resist the occurrence of mechanical damage to the tissue due to any external stimuli, while at the same time maintaining the transmission of optimal amounts of force through the tissue.

Periodontal ligament stem cells (PDLSCs) obtained from mature periodontal ligament (PDL) are a type of mesenchymal stem cells (MSCs) located in the perivascular space of the periodontium. They arise from the migratory neural crest cells from which the periodontal tissues arise. They have immunogenic and immunosuppressive abilities, which are comparable to that of the bone marrow MSCs [2]. MSCs are present in the ECM of most tissues in the human body, such as tendons (TEN), bone marrow, and adipose tissue. They have high self-renewal potential in addition to pluripotency, so they are more advantageous as compared to other forms of stem cells, such as embryonic stem cells, which pose high risks for the formation of teratoma [3]. The tendon tissue formed from the MSCs is cytologically and functionally of superior quality than the biological tendon tissue. MSCs show highly promising results for their use in the treatment of tendon-related injuries as they accelerate the tissue healing process in the tendon [4].

The tendon is a type of fibrous connective tissue that provides attachment between muscle and bone [5]. It has a high concentration of densely packed collagen fibres. Some of the most important components of the ECM of tendon tissue are the pluripotent stem cells. Tendon injuries are very common, especially in the field of sports and other similar physical activities. Chronic tendinopathy, tenderness, swelling, chronic pain on stimulation, and so on are frequently seen tendon-related complications. The crimp pattern of collagen fibres and the fibril size of the fibrotic scar tissue are smaller than that of the intact tendon and cannot return to their original mechanical strength for a long time after injury, causing significant dysfunction and disability [6]. One of the main challenges faced in the field of tendon repair is the unavailability of proper clinically relevant injury models where the injury and the repair are separated in time [7]. Injury models, particularly those related to wound healing, are a cogent application of self-organising tissues [8]. There needs to be more tendon injury models for degenerative anomalies. Tissue engineering or tissue regeneration is a very promising potential strategy for the treatment of tendon injuries or other tendon-related anomalies. Scaffolds are one of the most common strategies used in tendon tissue engineering as they provide mechanical support to the healing tissue until the new reparative matrix has been fully deposited, thus preventing re-injury [9].

The pluripotent property of the stem cells present in the ECM of the tendon makes stem cell therapy a promising strategy for tendon tissue regeneration. The use of PDLSCs as the stem cell source could be a potential new protocol for cell seeding in regenerative scaffolds.

Hyaluronic acid (HA) is a natural component of the ECM of most tissues in the human body. It is a biodegradable hydrophilic polysaccharide, which is composed of D-glucuronic acid and N-acetyl glucosamine linked by beta (1 → 4) interglycosidic linkages [10]. It has a hydrating and moisturising effect on tissues, so it is commonly used as a lubricant or coating material. Due to the presence of acidic non-sulphated glycosaminoglycans, hyaluronic acid maintains the physical form and viscoelasticity of the ECM [11]. For the same reason, it is used as a cell-seeding base for rigid or colloidal scaffolds and a carrier for numerous bioactive compounds. It is also being used as a ligand for cluster of differentiation 44 (CD44) [12]. Hyaluronic acid also has important applications in tissue engineering, tissue regeneration, drug delivery systems, and cosmetics [13].

*Ocimum sanctum L.* (OS), commonly known as Tulsi, the queen of herbs, is a traditional medicinal plant. It belongs to the family *Lamiaceae* of the order *Lamiales* of the class *Eudicots* of the division Angiospermae of the kingdom Plantae. It is an erect branched sub-shrub of around 30-60 cm in height with petiolate ovate leaves around 5 cm in length. The plant bears purplish flowers in elongated racemes in close whorls. Ayurveda describes it as an “Elixir of Life” because of its diverse medicinal properties [14]. It has been in use for a very long time as a home remedy for most common illnesses such as the common cold, fever, and so on. It is used as an expectorant, analgesic, and diaphoretic, and also as a general vitaliser because of its ability to increase physical endurance. It has high anti-inflammatory, anti-diabetic, anti-asthmatic, anti-cancer, antiemetic, anti-fertilitic, hepatoprotective, hypotensive, hypolipidemic, anti-stress, antimicrobial, and antioxidant activities [15].

The joint between the tooth and the alveolar bone in the alveolar socket is a type of structure called gomphosis. The mobile peg-and-socket joint allows only very minimal movement of the tooth into the socket during biting and/or chewing. Periodontal fibres run from the alveolar socket to the root of the tooth along with proprioceptive receptor nerve terminals forming a complex histostructure. The hierarchical architecture of soft tissue in between two hard tissues provides a challenge to tissue regeneration attempts using most types of scaffolds other than hydrogels, as the stiffness of the scaffold prevents it from properly adapting to the root surface construct. The topical application of hyaluronic acid hydrogel has been evaluated as a potential supplement in the treatment of gingivitis and chronic periodontitis, as it brought about a significant reduction in peroxidase and lysozyme activities. Hence, hyaluronic acid hydrogels are the most effective option for the treatment of periodontal defects by tissue regeneration method [16].

Previous research has been done using human adipose-derived stem cells and rabbit-derived stem cells with the ECM of tendon tissue and the viability, proliferation, and differentiation rates were recorded to be high [5,17,18]. The current methods of tissue engineering possess a lot of drawbacks such as the chances of improper neovascularization and the effect of the absorption kinetics of the scaffolds on their success rate [19]. Hence, we have directed our efforts into the development of a novel protocol of scaffold fabrication using hyaluronic acid hydrogels by the incorporation of herbal components to increase efficacy instead of chemical components, which may cause cytotoxicity.

## Materials and methods

This in vitro research was done to find out if the addition of the extract of a natural product, which in this case is *Ocimum sanctum*, to the ECM from tendon will increase the regenerative tenogenic potential of PDLSCs produced in hyaluronic acid scaffolds. The presence of the formed tendon cells was confirmed by the tenogenesis assay and the viability, biocompatibility, and structural and organisational properties of the scaffolds were analysed using scanning electron microscopy (SEM), swelling, compatibility, compression, and differentiation analyses. This was an original research conducted in a university scientific laboratory.

Preparation of plant extract

**Figure 1:**
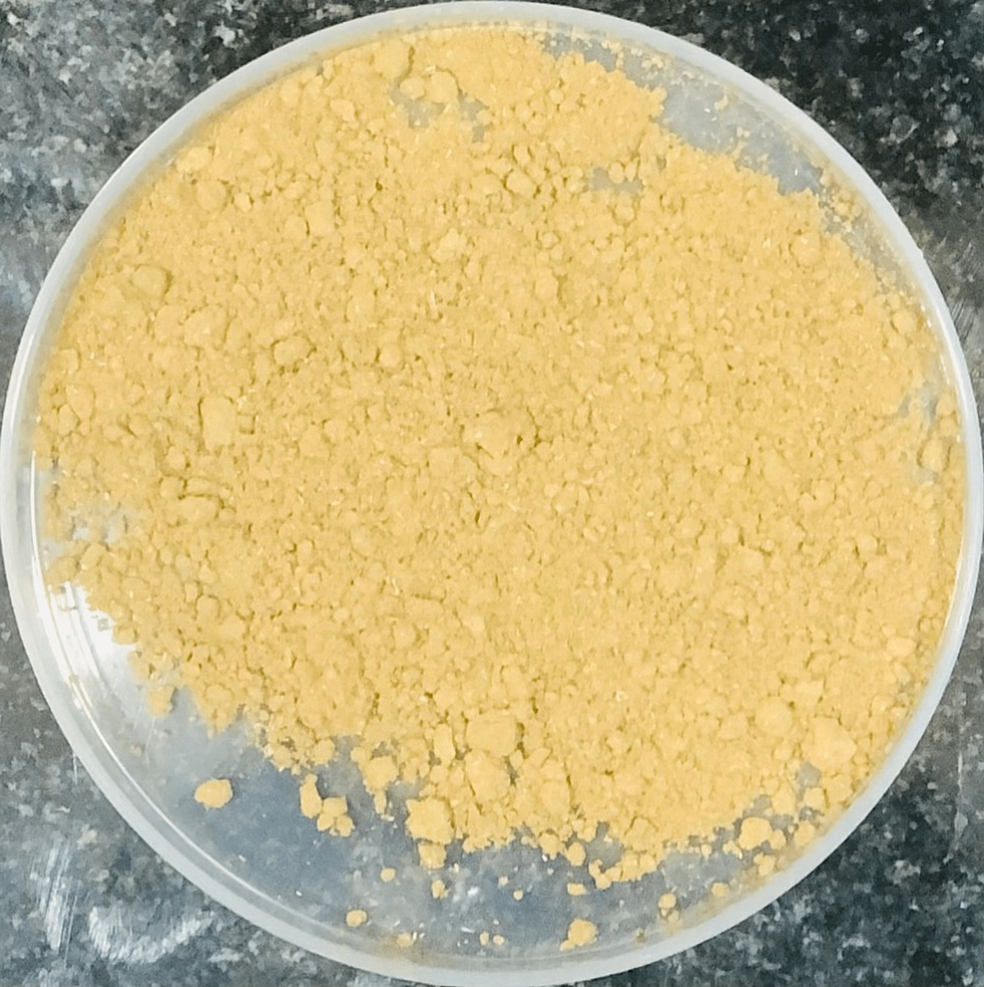
Powdered Ocimum sanctum (OS)

A fresh specimen of the leaves of the plant *Ocimum sanctum* was obtained from a herbal garden and confirmed by a botanist. The collected specimen was washed thoroughly with distilled water and air-dried in the shade to remove the moisture content. Then the leaf specimen was ground into fine powder using a mortar and pestle and stored away from moisture until use (Figure 1).

Extraction of secondary metabolites from *Ocimum sanctum*

**Figure 2:**
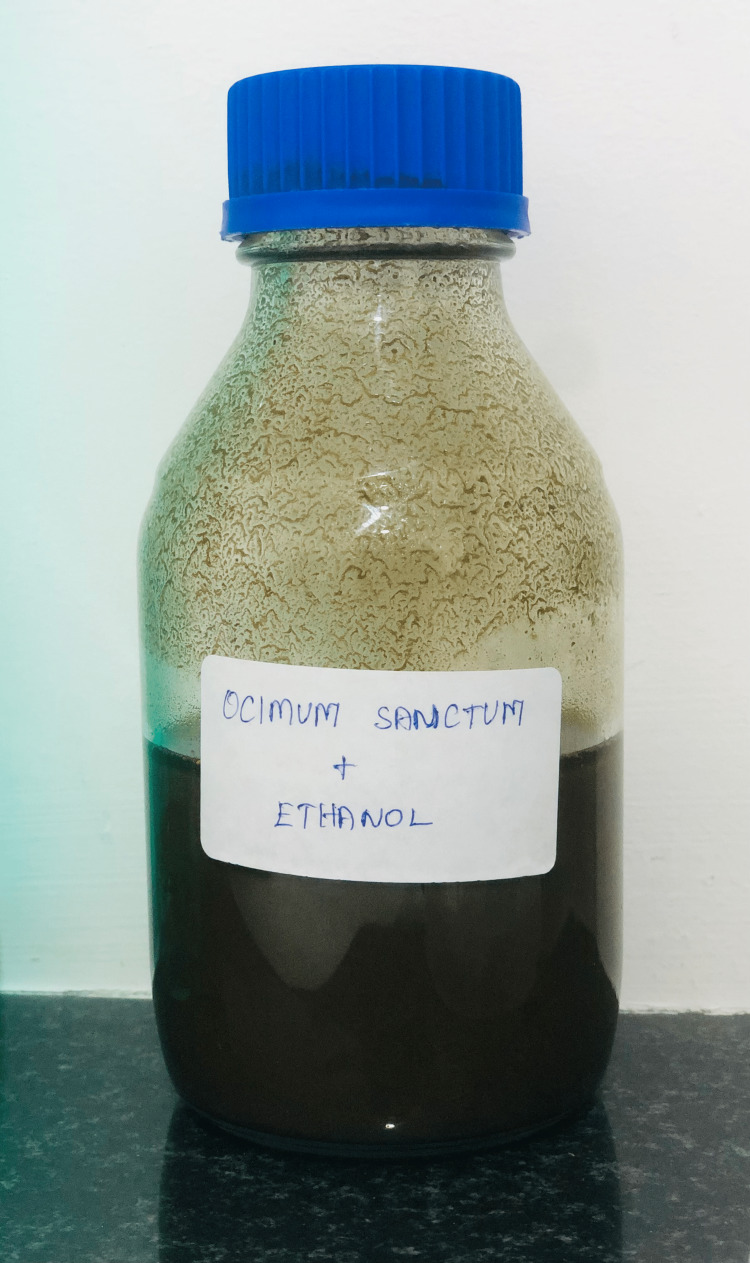
Ethanolic extract of Ocimum sanctum (OS)

Fifty grams of dried powdered *Ocimum sanctum* was added to 250 mL of ethanol and placed in a shaker for 24 hours at 120 rpm and then left to settle for 24 hours. The supernatant was collected and subjected to flash evaporation (Round Bath Evator, Equitron Medica Pvt. Ltd., Mumbai, India) to obtain the *Ocimum sanctum* extract (Figure 2).

Fabrication of methacrylated hyaluronic acid (HA-MA)

One gram of cosmetic-grade deproteinised synthetic hyaluronic acid was dissolved in 100 mL of water. The pH of the solution was adjusted to 8 using a 2N sodium hydroxide solution. A total of 7.89 mL of methacrylic anhydride was added to the hyaluronic acid solution and stirred for four hours. The pH was maintained at 8 throughout the reaction. The solution was passed through a dialysis tube with a 3000 mol. wt. cutoff for three days. The final material was lyophilised in a freeze dryer [20]. The freeze-dried samples were stored in a refrigerator until use.

Preparation of extracellular matrix from ovine tendon

Tendons were harvested in sterile condition from an ovine carcass obtained from a local slaughterhouse. The tendon was then reduced into minute fragments of dimensions 2 mm x 2 mm using a scalpel. The tendon fragments were then immersed in 20 mL of 10% phosphate-buffered saline (PBS) followed by decellularisation fluid. One gram of sodium dodecyl sulphate (SDS) was added, followed by 200 µL of Triton-X to 100 mL of distilled water to prepare the decellularisation solution. Then 25 mL of the decellularisation solution was added to the tendon sample and it was placed in the shaker at 37°C till a froth was formed. The froth was removed and the decellularisation solution was refilled every six hours for three days. The resulting decellularised tendon ECM was dried in a freeze dryer and stored in a refrigerator.

Hydrogel fabrication and study groups

The photocrosslinkable hydrogel samples were prepared by combining the methacrylated hyaluronic acid, the natural product *Ocimum sanctum*, and the ECM of the tendon, using the I2959 photocatalyst. The formulations were kept under ultraviolet light of wavelength 395 nm for five minutes to form hydrogels.

The study group hydrogels were HA (1% HA-MA), TEN (10 mg/mL of the ECM of the ovine tendon), HA_OS (1% HA-MA and 10 mg/mL of *Ocimum sanctum* extract), HA_TEN (1% HA-MA and 10 mg/mL of ECM of ovine tendon), and HA_OS_TEN (1% HA-MA, 10 mg/mL of *Ocimum sanctum* extract, and 10 mg/mL of ECM of ovine tendon).

Scanning electron microscopy analysis

Overnight, the hydrogel samples were fixed in a 4% paraformaldehyde solution. The samples were then dehydrated using a series of graded ethanol baths from 10% to 100%. After being fixed in aluminium stubs, the dehydrated samples were subsequently coated with gold using a sputter coater at 37°C. The samples were then photographed and inspected using a scanning electron microscope (JEOL JSM-IT800 FE-SEM, JEOL Ltd., Tokyo, Japan).

Swelling analysis

Some of the hydrogel samples were dry weighed and then immersed in 5 mL of 10% PBS solution at 37°C. After an hour, the samples were removed from the PBS and dabbed with a Kimwipe to remove any excess fluid on the surface. The samples were then wet-weighed to test the level of swelling and fluid absorption. The swelling ratio (SR) was calculated by the following equation using dry weight (W0) and wet weight (Ww) as shown: SR = ((Ww - W0)/W0) x 100%. Data were presented as mean ± standard deviation, where n = 3.

Mechanical compression analysis

The mechanical strength of the samples was tested by subjecting the samples to a wide range of compressional forces to analyse their behaviour under high and low stresses. The samples were tested for the required force to cause fracture using a universal testing machine (Instron ElectroPuls E3000, Instron Corp., Norwood, MA). The compressive force was selected to be applied at a cross-head speed of 1 mm/min until fracture. A piece of tin foil of thickness 0.1 mm was placed between the loading piston and the hydrogel sample. The hemispherical steel head indenter of diameter 5 mm was placed on the surface of the hydrogel sample. The value of the maximum force necessary to cause a fracture was recorded in newtons (N) and the compressive stress was recorded in megapascals (MPa) [21]. Data were presented as mean ± standard deviation, where n = 3.

Cell culture of PDLSCs

At Saveetha Dental College and Hospitals (SDC), five individuals between the ages of 18 and 35 were chosen based on their general health and elective surgical operations, according to the authorised standards of the SDC Institutional Review Board. Disease-free impacted third molars were extracted from these individuals. The teeth were stored in Hank’s balanced salt solution (HBSS) to preserve cell viability. PDL cells were scraped from the radicular portions of the teeth below the cementoenamel junction and then enzymatically digested for one hour at 37°C in a solution of 3 mg/mL collagenase type I (RM2075 Collagenase Type I, HiMedia Labs Ltd., Chennai, India) and 4 mg/mL dispase (Dispase, HiMedia Labs Ltd., Chennai, India). A 70 m cell strainer (Cell Strainer, HiMedia Labs Ltd., Chennai, India) was used to separate PDL cells from several persons into single-cell suspensions. The samples were centrifuged at 400 g for 10 minutes [22] and then resuspended in Dulbecco's Modified Eagle's Medium (DMEM) (12-604F DMEM, Lonza Group AG, Basel, Switzerland) supplemented with 10% FBS (HiMedia RM9955 FBS, HiMedia Labs Ltd., Chennai, India) and 1% penicillin-streptomycin (PenStrep). Following two passages, 10,000 cells were seeded into each well of a 48-well plate to conduct compatibility, differentiation, and tenogenesis analyses.

MTT compatibility assay

The cell viability and proliferation levels were tested by the 3-(4,5-dimethylthiazol-2-yl)-2,5-diphenyl-2H-tetrazolium bromide (MTT) assay. The samples were seeded in a 96-well plate with 100 µL per well. MTT solution (10 µL) was added and the solution was incubated at 37°C for three hours. A total of 100 µL of solubilisation solution of dimethyl sulphoxide (DMSO) was added to each well to dissolve the crystalline formazan product formed. The absorbance was recorded at 570 nm. Data were presented as mean ± standard deviation, where n = 3.

Differentiation analysis

The hydrogel scaffolds were placed in 24-well plates coated with 0.3% poly (2-hydroxyethylmethacrylate) (polyHEMA) to make sure that the cells do not adhere to the polystyrene [23]. The scaffolds were incubated at 37°C and 5% CO2 for seven days. A conditioned cellular induction medium (2 mL) consisting of DMEM (12-604F DMEM, Lonza Group AG, Basel, Switzerland) supplemented with 10% foetal bovine serum (FBS) (HiMedia RM9955 FBS, HiMedia Labs Ltd., Chennai, India) and 1% penicillin-streptomycin (PenStrep) was added to each well [24]. The differentiation rates were visualised at regular intervals using a biological microscope (Labomed Microscope, Labomed Inc., Los Angeles, CA).

Tenogenesis assay by Sirius red staining

The conditioned cellular induction medium was withdrawn after seven days of cellular induction, and the cells were then rinsed with PBS. The cells were then washed thrice with distilled water after being fixed with 70% ethanol for 30 minutes. With 0.1% picrosirius red (Sirius red in a saturated aqueous solution of picric acid) (Picrosirius Red, Sigma-Aldrich, St. Louis, MO), the deposited collagen was dyed. The stain was solubilized with 0.5 mL of 1:1 (vol/vol) 0.1% sodium hydroxide and absolute methanol for 30 minutes at room temperature to quantify the stained nodules. A 96-well plate was filled with 0.1 mL of the solubilized dye, and the absorbance was measured at 540 nm. Data were shown as mean standard deviation, with n = 3.

Statistical analysis

All the experiments were conducted in triplicate (n = 3). For each experiment, five samples of each material were used. Data were presented as mean ± standard deviation to minimise error, and considering the parametric nature of data, one-way analysis of variance (ANOVA) was performed to derive inferential statistics.

## Results

Scanning electron microscopy analysis

**Figure 3:**
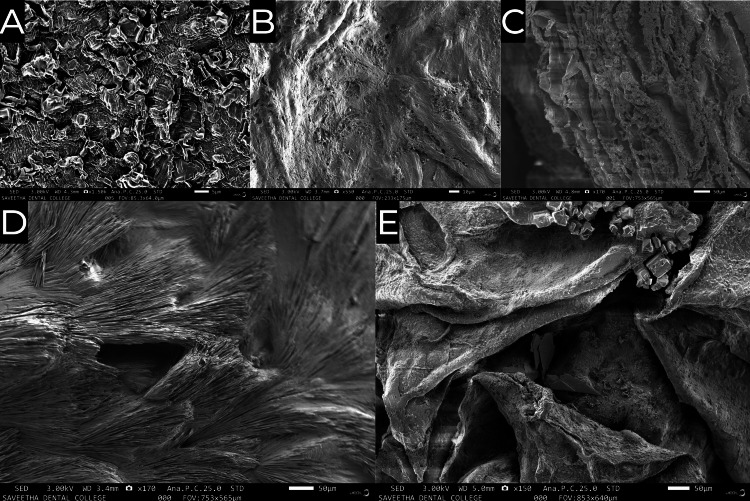
Scanning electron microscope image of (A) hyaluronic acid (HA), (B) tendon (TEN), (C) hyaluronic acid + tendon (HA_TEN), (D) hyaluronic acid + Ocimum sanctum (HA_OS), and (E) hyaluronic acid + Ocimum sanctum + tendon (HA_OS_TEN).

The characteristics of the molecular morphology of the hydrogel samples were visualised by SEM, as shown in Figure 3.

Hyaluronic acid was shown to have a very rough morphology with a high degree of cresting and troughing (Figure 3A). The ECM of the tendon had a fibrous morphology with a very high density of interconnected fibres (Figure 3B). The hyaluronic acid + ECM of the tendon sample had a lower degree of cresting and troughing than the hyaluronic acid sample owing to the slight amount of layering or folding of the surface morphology (Figure 3C). The hyaluronic acid + *Ocimum sanctum* sample also had a fibrous structure, but in this sample, the fibres were arranged linearly and were oriented more or less in the same direction with only slight variations in the angulation (Figure 3D). The hyaluronic acid + ECM of the tendon + *Ocimum sanctum* sample had a very rough structure with a high degree of cresting and troughing accompanied by an interconnected fibrous network with the presence of layering and folding as well (Figure 3E).

Swelling analysis

**Figure 4:**
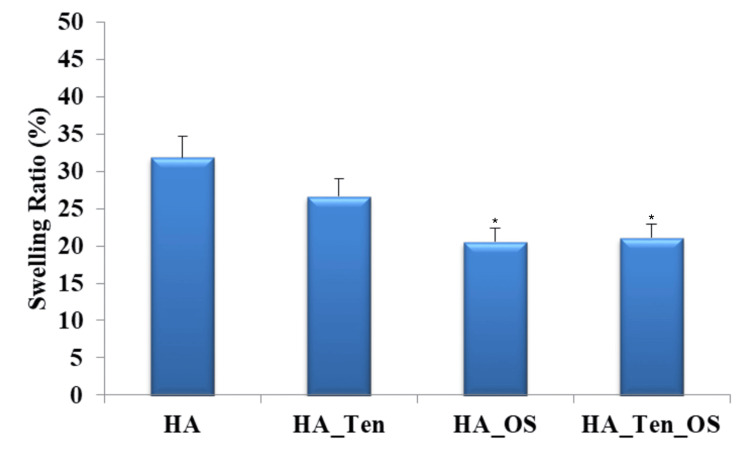
Swelling analysis The standard deviation in each case is 0.05 (HA), 0.05 (HA_Ten), 0.04* (HA_OS), and 0.04* (HA_Ten_OS). * denotes a p-value less than 0.05, which is considered statistically significant. HA: hyaluronic acid; HA_Ten: hyaluronic acid + tendon; HA_OS: hyaluronic acid + *Ocimum sanctum*; HA_Ten_OS: hyaluronic acid + tendon + *Ocimum sanctum*.

The amount of fluid absorbed or imbibed by each sample was calculated and the values were compared, as shown in Figure 4.

The HA sample showed 32% swelling, the HA_TEN sample showed 27%, the HA_OS sample showed 20%, and the HA_OS_TEN sample showed 23% swelling. Thus, the swelling ratio was least in the case of the HA_OS sample, closely followed by the HA_OS_TEN sample.

Mechanical compression analysis

The mechanical strength of the samples was analysed by subjecting them to a range of compressive forces and their compressive limits were recorded. The values were compared, as shown in Table 1.

**Table 1:** Mechanical compression analysis A p-value less than 0.05 is considered statistically significant. HA_OS: hyaluronic acid + *Ocimum sanctum*; HA_TEN: hyaluronic acid + tendon; HA_OS_TEN: hyaluronic acid + *Ocimum sanctum* + tendon.

No.	Specimen label	Maximum force (N)	Compressive stress at maximum force (MPa)	p-value
1.	HA_OS	14.46	0.18	0.03
2.	HA_TEN	108.72	1.38	0.04
3.	HA_OS_TEN	8.73	0.11	0.04

The HA_OS sample withstood 0.18 MPa pressure at a maximum force of 14.46 N. The HA_TEN sample withstood 1.38 MPa pressure at a maximum force of 108.72 N. The HA_OS_TEN sample withstood 0.11 MPa pressure at a maximum force of 8.73 N. The compressive stress at the maximum force withstood before breaking was highest for the HA_TEN sample, followed by the HA_OS_TEN sample.

MTT compatibility analysis

**Figure 5:**
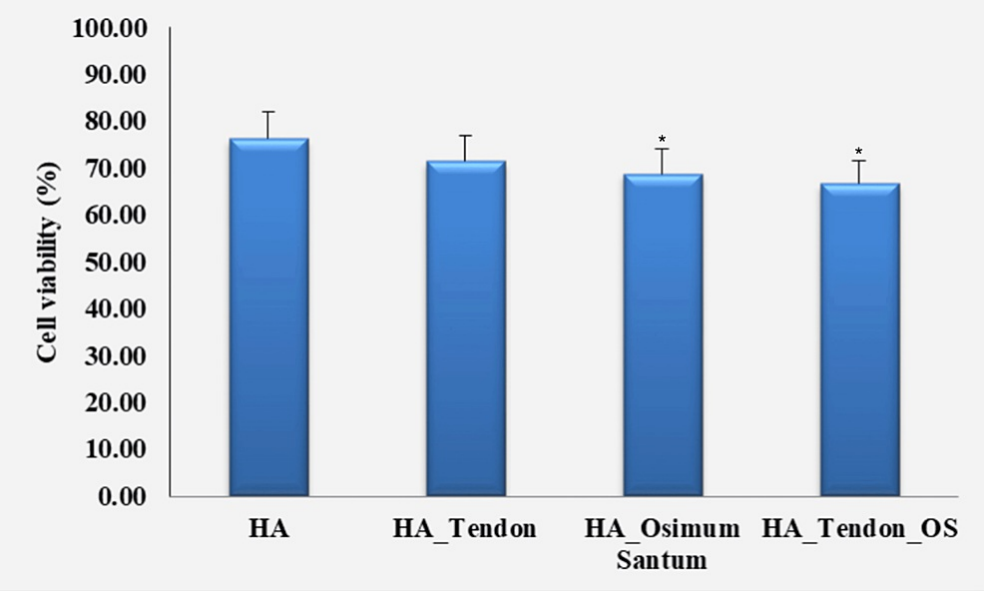
Cell viability analysis by MTT compatibility assay The standard deviation in each case is 0.05 (HA), 0.05 (HA_Ten), 0.04* (HA_OS), and 0.04* (HA_Ten_OS). * denotes a p-value less than 0.05, which is considered statistically significant. HA: hyaluronic acid; HA_Ten: hyaluronic acid + tendon; HA_OS: hyaluronic acid + *Ocimum sanctum*; HA_Ten_OS: hyaluronic acid + tendon + *Ocimum sanctum*; MTT: 3-(4,5-dimethylthiazol-2-yl)-2,5-diphenyl-2H-tetrazolium bromide.

The viability rates of the PDLSCs in the samples were analysed by the MTT compatibility assay and the values were compared, as shown in Figure 5.

The HA sample showed 75% cell viability, the HA_TEN sample showed 70%, the HA_OS sample showed 68%, and the HA_OS_TEN sample showed 67% viability. Thus the MTT assay showed similar values of cell viability for all the samples in the range of 65%-75%.

Differentiation analysis

**Figure 6:**
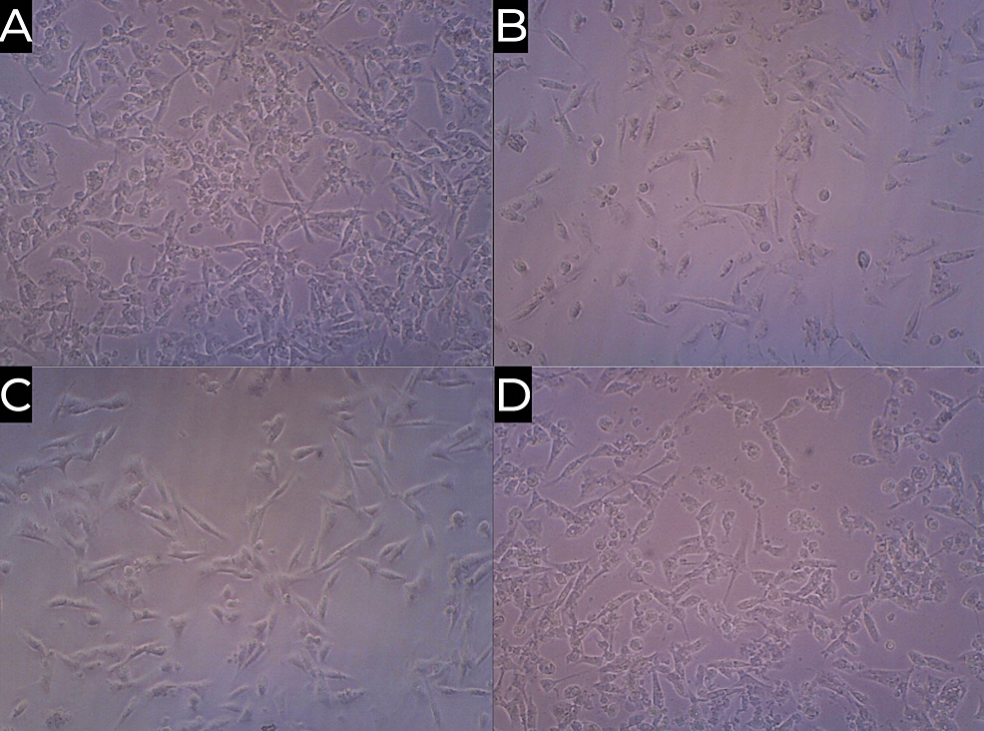
Differentiation analysis of (A) hyaluronic acid (HA), (B) hyaluronic acid + tendon (HA_TEN), (C) hyaluronic acid + Ocimum sanctum (HA_OS), and (D) hyaluronic acid + Ocimum sanctum + tendon (HA_OS_TEN)

The formation of tendon tissue from the PDLSCs was visualised at 20x magnification for each sample, as shown in Figure 6.

The maximum rate of cell differentiation was observed in the case of the HA sample, as shown in Figure 6A, followed by the HA_OS_TEN sample, as shown in Figure 6D.

Tenogenesis assay by Sirius red staining

**Figure 7:**
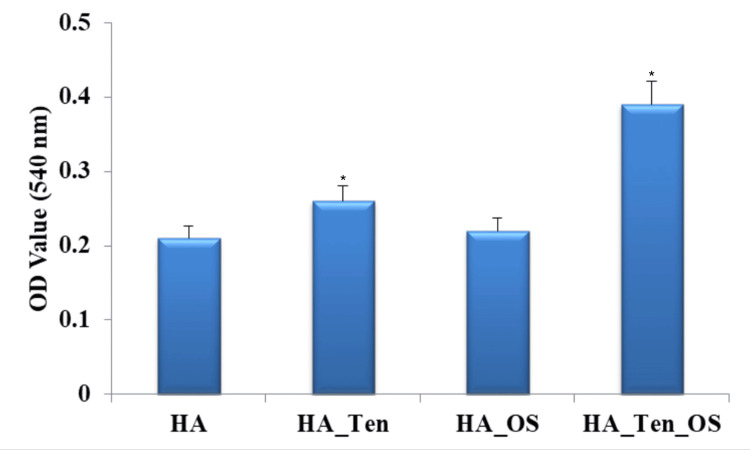
Tenogenesis assay by picrosirius red staining The standard deviation in each case is 0.05 (HA), 0.04* (HA_Ten), 0.05 (HA_OS), and 0.04* (HA_Ten_OS). * denotes a p-value less than 0.05, which is considered statistically significant. HA: hyaluronic acid; HA_Ten: hyaluronic acid + tendon; HA_OS: hyaluronic acid + *Ocimum sanctum*; HA_Ten_OS: hyaluronic acid + tendon + *Ocimum sanctum*.

The tenogenic potential of the PDLSCs present in the study groups was analysed by picrosirius red staining and the values were compared, as shown in Figure 7.

The HA sample showed 42% tenogenesis, the HA_TEN sample showed 52%, the HA_OS sample showed 46%, and the HA_OS_TEN sample showed 80% tenogenesis. The highest rate of tenogenesis was shown by the PDLSCs present in the HA_OS_TEN sample, followed by the HA_TEN sample.

## Discussion

Various synthetic scaffolds are currently available and in use for regenerative purposes, but the use of natural materials in the fabrication of hydrogel scaffolds offers higher biocompatibility and lower cytotoxicity, hence it is preferred to synthetic hydrogels. Although there are many studies being conducted and reported on the use of hydrogel scaffolds for periodontal regeneration [25], studies are scarce on the use of natural products and ECM of the tendon in hyaluronic acid hydrogels. This study was conducted to find a suitable natural product that can be incorporated into hyaluronic acid hydrogel scaffolds to increase the regenerative potential of the PDLSCs. We chose the traditional medicinal plant *Ocimum sanctum* as the natural product and the results were very promising.

The scanning electron micrographs of the samples were obtained to study the morphology of the samples and examine and compare the changes that occur when the raw materials and materials are taken in conjunction with each other. From Figure 3A, we observe that the hyaluronic acid has a very rough morphology with a high degree of cresting and troughing while from Figure 3B, we observe that the ECM of the tendon has a fibrous morphology with a very high density of interconnected fibres. On taking both the materials in conjunction, as shown in Figure 3C, we observe that the hyaluronic acid + ECM of the tendon sample has a lower degree of cresting and troughing than the hyaluronic acid sample, owing to the slight amount of layering or folding of the surface morphology. Further, in Figure 3D, we observe that the hyaluronic acid + *Ocimum sanctum* sample also has a fibrous structure, but in this sample, the fibres are arranged linearly and are oriented more or less in the same direction with only slight variations in the angulation. Finally, in Figure 3E, we observe that the hyaluronic acid + ECM of tendon + *Ocimum sanctum* sample has a very rough structure with a high degree of cresting and troughing, accompanied by an interconnected fibrous network with the presence of layering and folding as well. The highly irregular structure achieved by using all the materials in conjunction increases the surface area of the hydrogel scaffolds and thus contributes to increasing the interaction between the scaffold and the surrounding tissues at the placement site. The increased interaction will provide increased mechanical stability for the scaffold while it regenerates the damaged or lost tendon tissue so that there is no re-injury while the healing process is in progress [9].

Swelling ratio analysis is performed to calculate the extent to which the hydrogel scaffold prepared will expand on the absorption of the tissue fluid surrounding the site of its placement. The swelling ratio optimally should be low so that the hydrogel sample does not expand in tissue fluid and damage the surrounding tissues or disrupt the functional working of the surrounding or underlying tissue structures. From Figure 4, we see that the least swelling ratio is possessed by the sample containing hyaluronic acid and *Ocimum sanctum* at 20% (approx.) swelling, followed by the sample containing hyaluronic acid, ECM of the tendon, and *Ocimum sanctum* at 21% (approx.) swelling. We see that the addition of *Ocimum sanctum* decreases the swelling ratio to around 20%. This proves that the addition of the natural product *Ocimum sanctum* decreases the swelling rate and increases the effectiveness of the hyaluronic acid hydrogel scaffolds with the ECM of the tendon. These results are better than the results obtained from previous research [26].

The scaffolds are subjected to mechanical compressional analysis to test the mechanical strength and physical durability of the scaffold samples to ensure that the scaffolds do not suffer damage by physical activity or external impact on the body part where it is placed. From Table 1, we see that the maximum compressive stress is developed at maximum force application before fracture in the sample containing hyaluronic acid and ECM of the tendon (1.38 MPa at 108.72 N), followed by the sample containing hyaluronic acid and *Ocimum sanctum* (0.18 MPa at 14.46 N) and the sample containing hyaluronic acid, ECM of the tendon, and *Ocimum sanctum* (0.11 MPa at 8.73 N). Although the mechanical tolerance of the sample containing hyaluronic acid, ECM of the tendon, and *Ocimum sanctum* is not higher than that of the other samples, it still has a very significant mechanical tolerance toward compressional forces or other physical stimuli. This proves that the addition of the natural product *Ocimum sanctum* to the hyaluronic acid hydrogel scaffolds with the ECM of the tendon provides a very strong and durable scaffold for tendon regeneration. These results are better than the results obtained from previous research [26].

The cell viability of the hydrogel samples is assessed by the MTT compatibility assay to evaluate the efficacy, suitability, and biocompatibility of the scaffolds for tissue regeneration in the human body. From Figure 5, we see that the MTT assay shows the highest degree of cell viability in the sample containing hyaluronic acid at 75% (approx.), followed by the sample containing hyaluronic acid and ECM of the tendon at 70% (approx.), the sample containing hyaluronic acid and *Ocimum sanctum* at 68% (approx.), and the sample containing hyaluronic acid, ECM of tendon, and *Ocimum sanctum* at 65% (approx.). Although the cell viability level of the sample containing hyaluronic acid, ECM of the tendon, and *Ocimum sanctum* is not higher than that of the other samples, it still has a very significant cell viability rate of 65% (approx.). This proves that the addition of the natural product *Ocimum sanctum* to the hyaluronic acid hydrogel scaffolds with the ECM of the tendon provides a very viable and biocompatible scaffold for tendon regeneration. These results are better than the results obtained from previous research [27].

The differentiation analysis is done to visualise the number of tendon cells formed from the PDLSCs in the hyaluronic acid hydrogel scaffold samples. From Figure 6A, we see that the maximum formation of tendon cells is seen in the sample containing hyaluronic acid, followed by the sample from Figure 6B containing hyaluronic acid, ECM of the tendon, and *Ocimum sanctum*. On comparing the hyaluronic acid + ECM of the tendon + *Ocimum sanctum* with the hyaluronic acid + ECM of the tendon from Figure 6C and hyaluronic acid + *Ocimum sanctum* from Figure 6D, we see that there is more tendon cell formation in case of the hyaluronic acid + ECM of tendon + *Ocimum sanctum* sample. This proves that the addition of the natural product *Ocimum sanctum* in conjunction with ECM of the tendon will provide a hyaluronic acid hydrogel scaffold with very high tenogenic potential. These results are better than the results obtained from previous research [28].

Picrosirius red staining reveals the enhancing tenogenic activity of *Ocimum sanctum* on the PDLSCs by calculating the absorbance (optical density (OD) value) at 540 nm. From Figure 7, we see that the maximum number of tendon cells are formed in the sample containing hyaluronic acid, ECM of the tendon, and *Ocimum sanctum*, which has an OD value of 0.4 (approx.) at 540 nm. It is closely followed by the sample containing hyaluronic acid and ECM of the tendon, which has an OD value of 0.26 (approx.) at 540 nm. On comparing these two values, we see that the addition of *Ocimum sanctum* has increased the OD value to almost 1.5x. This proves that the addition of the natural product *Ocimum sanctum* will increase the tenogenic potential of the PDLSCs in the hyaluronic acid hydrogel scaffold with the ECM of the tendon.

From this study, we see that the hyaluronic acid hydrogel scaffold prepared in conjunction with *Ocimum sanctum* and the ECM of the tendon can be viably used for tissue regeneration. This can be used for various restorative applications, especially in periodontal regeneration considering the efficacy of hyaluronan hydrogels in the treatment of chronic periodontitis. Since this is only an in vitro study, we need to conduct animal trials and perform detailed investigations into the suitability of using these hydrogels as a safe treatment modality for the treatment of periodontal defects and anomalies [16].

There are several limitations to this study, as it is a very preliminary in vitro study on the combination of the natural product *Ocimum sanctum* with PDLSCs from the ECM of the tendon in a hyaluronic acid hydrogel. The swelling rates and the mechanical tolerance levels of the hyaluronic acid + ECM of the tendon + *Ocimum sanctum* sample obtained in this study are not higher than the control samples of HA, TEN, OS, HA_TEN, and HA_OS, even though they are optimal. The MTT assay shows the lowest value of cell viability and biocompatibility for the hyaluronic acid + ECM of the tendon + *Ocimum sanctum* sample when compared to the control samples.

Further research needs to be done into identifying other natural or synthetic products that can be used in conjunction with *Ocimum sanctum* and ECM of the tendon in hyaluronic acid. Investigations need to be conducted into whether the active compounds present in *Ocimum sanctum* have affected the results of the tests that have been conducted in this study. More combinations should be investigated to further optimise the swelling rates and mechanical tolerance and increase the cell viability and biocompatibility of the hydrogel scaffolds.

## Conclusions

The results obtained in this study conclusively prove that the addition of the natural product *Ocimum sanctum* to mesenchymal stem cells of the extracellular matrix of tendon increases the tenogenic potential of the stem cells. Thus, this fabrication is a suitable material that can be incorporated into hydrogel scaffolds to increase the regenerative potential of PDLSCs for tenogenic purposes. *Ocimum sanctum* is a very common natural product, which is readily available in most countries of the world, and PDLSCs are easily extractable from human teeth and show high immunomodulatory activity, which is beneficial in speeding up wound healing and regeneration. This opens up various prospects to use the hyaluronic acid scaffolds with *Ocimum sanctum,* which contains PDLSCs as a means of reparative therapy in tendon-related injuries and maladies.
